# Evaluation of the Capacity of PCR and High-Resolution Melt Curve Analysis for Identification of Mixed Infection with *Mycoplasma gallisepticum* Strains

**DOI:** 10.1371/journal.pone.0126824

**Published:** 2015-05-13

**Authors:** Seyed A. Ghorashi, Anna Kanci, Amir H. Noormohammadi

**Affiliations:** 1 Asia Pacific Center for Animal Health, Faculty of Veterinary and Agricultural Sciences, The University of Melbourne, Werribee, Victoria, 3030, Australia; 2 Asia-Pacific Centre for Animal Health, Faculty of Veterinary and Agricultural Sciences, The University of Melbourne, Parkville, Victoria, 3010, Australia; GI Lab, UNITED STATES

## Abstract

Pathogenicity and presentation of *Mycoplasma gallisepticum* (MG) infection may differ from one strain to another and this may have implications on control measures. Infection of individual birds with more than one MG strain has been reported. A PCR followed by high resolution melt (HRM) curve analysis has been developed in our laboratory and routinely used for detection and differentiation of MG strains. However the potential of this test for identification of MG strains in a mixed specimen has not been evaluated. In the present study, the capability of PCR-HRM curve analysis technique, targeting *vlhA* and *pvpA* genes was assessed for identification of individual MG strains in a mixed population. Different DNA ratios of two MG strains from 1 to 10^-4^ ng were tested with some generated conventional and normalized curves distinct from those of individual strains alone. Using genotype confidence percentages (GCP) generated from HRM curve analysis, it was found that *vlhA* PCR-HRM was more consistent than *pvpA* PCR-HRM for the detection of MG ts-11 vaccine strain mixed with any of the MG strains 6/85, F, S6 or a field isolate. The potential of *vlhA* PCR-HRM to detect mixed MG strains in a specimen was found to be primarily dependent on quantity and proportion of the target DNAs in the mixture. This is the first study examining the capacity of PCR-HRM technique for identification of individual MG strains in a mixed strain population.

## Introduction

*Mycoplasma gallisepticum* (MG) is an important poultry pathogen causing economic loss in many parts of the world. Eradication policy in chicken farms is the preferred method of MG control; however, vaccination is used in areas when eradication is not economically feasible [1].

Isolation of more than one *Mycoplasma* species from a single site of one host has been reported [2, 3]. A number of earlier reports have also indicated that birds vaccinated with a live MG vaccine and experimentally challenged with a field strain may harbour both strains at the same time [4–7].

Identification of MG strains/isolates involved in an outbreak is important for the subsequent control measures taken. Some MG isolates from wild birds can pose risks to poultry flocks and cause potential problems for MG control [8]. It is reported that chickens infected with MG through contact with infected house finches did not develop clinical disease, gross or microscopic lesions [9] while Mycoplasma infection in turkeys caused by low pathogenic house finch isolate was found to be mild [10]. Therefore, MG strain/isolate involved in an outbreak may influence the clinical and control approaches. A number of molecular diagnostic methods have been reported for differentiation of individual MG strains/isolates [11–16]. However, limited reports are available for detection and differentiation of individual strains in a mixed strain infection. Conventionally, detection of individual MG strains in a mixed infection is carried out using culture and cloning. However, this might be difficult as faster growing strains may overgrow the other strain in the culture [17] making it difficult to detect the slower growing strain. Mixture of two *Mycoplasma* species have been detected by using immunofluorescence [3, 17] or immunofluorescence combined with immunoperoxidase [18]. Differentiation between two MG strains in a mixed infection has also been made based on their antibiotic sensitivity [19]. These techniques however require culture of the organism that is time consuming and carry the risk of dominant strain overgrowing the other strain. The mixtures of the live vaccine strain ts-11 and a field isolate have also been successfully detected using PCR-RFLP [20]. However, this technique requires, gel purification of PCR-amplified DNA, enzymatic digestion of DNA and gel electrophoresis which are time consuming and relatively expensive. Real-time PCR has been used for differentiation of MG vaccine and challenge strains using specific probes [16]. However, strain differentiation was restricted only to combination of known strains and therefore MG strains with different sequences to the target strains may not be differentiated.

We have recently compared the differentiation power of PCR-HRM curve analysis using *vlhA*, *pvpA*, *mgc2*, *gapA* genes and 16S-23S rRNA intergenic spacer region (IGSR) for differentiation of individual MG strains/isolates and found that PCR-HRM curve analysis using *vlhA* and *pvpA* have higher capacity than those of *mgc2*, *gapA and* IGSR for differentiation of MG strains [21]. The aim of this study was to assess the potential of PCR-HRM curve analysis of *vlhA* and *pvpA* genes for rapid and reliable detection of mixed MG strains.

## Materials and Methods

### Ethics statement

The clinical samples were collected from experimentally inoculated/vaccinated turkeys and the study was reviewed and approved by Animal Experimentation Ethics Committee (AEEC) at Melbourne University (permit 1212364.1).

### MG strains

MG vaccine strains ts-11, F and 6/85 were cultured or harvested from a commercial vial of vaccine (Table 1). The S6 strain and an Australian MG field isolate (86026) were cultured in Mycoplasma broth (MB) as described before [22]. The MG strains were selected from different MG genotypes [21] to evaluate and compare the discriminatory power of PCR-HRM.

**Table 1:** *Mycoplasma gallisepticum* cultures used in this study and their origin.

Strain/Isolate	Country of Origin	Reference	GenBank Acc No.	
			*vlhA*	*pvpA*
ts-11	Australia	[31]	FJ654144	JN001166
F	USA	[32]	FJ654142	JN001167
S6	USA	[33]	FJ654143	JN001168
6/85	USA	[34]	FJ654146	JN001169
86026	Australia	This study	JN001165	JN001170

In order to examine the potential of the newly developed assay for detection of mixed MG infection directly in clinical samples, three choanal swabs from experimentally inoculated mycoplasma-free field turkeys known to harbor field strain AP3As [23] and also a choanal swab from a 7 weeks old SPF chicken vaccinated with ts-11 were used in this study.

### DNA extraction

Total genomic DNA was extracted from mycoplasma cultures, from a commercial vial of vaccines, or from choanal swabs (see above) using DNA extraction kit (QIAGEN) according to the manufacturer’s instructions. Briefly, 0.5 ml of mycoplasma culture was pelleted by centrifugation at 20000 ***g*** for 5 min and the cell pellet was washed twice in phosphate buffered saline (PBS) and resuspended in 500 μl RLT lysis buffer (QIAGEN) and incubated for two hours at room temperature or overnight at 4°C. For clinical specimens, choanal swabs were placed in 500 μl RLT lysis buffer and incubated as described above. Then 15 μl of Qiaex II matrix (QIAGEN) and 300 μl 70% ethanol were added and mixed, and the lysate was loaded into a multispin MSK-100 column (Axygen Inc., Hayward, CA, USA), centrifuged for 30 s at 10000 ***g*** and the flow-through discarded. The column was washed with 600 μl RW1 buffer (QIAGEN) and twice with 500 μl RPE buffer (QIAGEN) and subjected to centrifugation at 18000 ***g*** for 90 s. The DNA was eluted from the matrix using 50 μl distilled water and the quantity of extracted DNA was measured by spectrophotometer and adjusted to 1 ng/μl for each specimen. Further DNA dilutions were prepared in dH_2_O if required before PCR or stored at -20°C for future use.

### PCR and High-resolution melt curve acquisition and analysis

Two sets of primers for *vlhA* [13] and *pvpA* [24] PCRs, were used as described in the respective reports. In order to assess the capability of PCR-HRM technique to identify mixed population of two different MG strains in a single specimen, the ts-11 vaccine strain was used as a model and different combinations of ts-11 vaccine strain with a second MG strain were tested in separate PCRs. Initially, in a series of PCR, the DNA concentration for ts-11 vaccine strain was kept constant but a series of 10 fold dilutions of DNA from the second MG strain (F, 6/85, S6 strain or 86026 field isolate) was added. In a second series of PCRs, the DNA concentration of contaminant MG strain was kept constant and a series of 10 fold dilutions of ts-11 DNA was added.

PCR amplifications were performed in 25 μl reaction volume on an I-Cycler thermal cycler (Bio-Rad). The reaction mixture contained 2 μl of genomic DNA (1 μl from each MG strain), 25 μM of each primer, 1.5 mM MgCL_2_, 1250 μM of each dNTP, 5 μM SYTO 9 green fluorescent nucleic acid stain (Invitrogen), 1× GoTaq Green Flexi Reaction buffer (Promega) and 1 U of Go Taq DNA polymerase (Promega). PCR condition for *vlhA* gene was one cycle of 94°C for 60 s, 40 cycles of 94°C for 10 s, 50°C for 10 s and 72°C for 10 s, and a final cycle of 72°C for one min. The optimal PCR conditions for amplification of *pvpA* gene was determined to be one cycle of 94°C for 2 min, 40 cycles of 94°C for 30 s, 55°C for 30 s and 72°C for 30 s, and a final cycle of 72°C for 10 min.

HRM curve analysis was performed in a Rotor-Gene 6000 thermal cycler (Corbett Life Science Pty Ltd). The PCR products were subjected to two different rampings of 0.2°C and 0.3°C/s between 70°C and 90°C. All specimens were tested in triplicates and their melting profiles analysed using Rotor Gene 1.7.27 software and the HRM algorithm provided. The normalization regions of 74.0–75.0 and 80.0–81.0 for *vlhA*-PCR, and of 81.0–82.0 and 89.0–90.0 for *pvpA*-PCR were used for analysis of amplicons.

### Comparison of HRM curves using genotype confidence percentage cut off point

Each MG strain (ts-11, S6, F, 6/85 and 86026) was set as ‘genotype’ and the HRM genotype confidence percentage (GCP) for the replicates of each MG strain was predicted by the software. Then the GCPs for each MG strain were averaged and the standard deviation (SD) was calculated. The cut off point and SD values were used to establish the GCP range for each of the MG strains in *vlhA* and *pvpA* genes. These GCPs were applied to HRM analysis to evaluate the discrimination power of the test to differentiate specimens containing a single MG strain and mixed MG strains.

Detection of mixed MG strains in comparison with un-mixed strains was assessed based on the average HRM-GCP values with any GCP of less than cut off point was regarded as ‘variation’ or mixed specimen.

In order to evaluate the capacity of PCR-HRM in detection of mixed infection in clinical specimens, equal concentrations of DNA (1ng) extracted from choanal swabs collected from experimentally inoculated birds with ts-11 or AP3AS MG strain, were mixed and tested.

## Results

### Cut off points were determined for each MG strain in the vlhA and pvpA PCR-HRM curve analysis

The mean of GCP and SD values for the MG strains/isolates (ts-11, 6/85, F, S6 and 86026) in *vlhA* and *pvpA* genes were calculated using each strain as reference. It is notable that the GCP and SD values for each MG strain represent the mean and variations of GCP when a GIVEN MG strain tested over a number of times in different runs, and these figures are not generated as a result of comparison between multiple MG strains. In the *vlhA* PCR-HRM, the GCP ± SD value when ts-11, 6/85, F, S6 and 86026 used as reference was 94.8 ± 7.5, 94.4 ± 5.7, 92.2 ± 10.7, 94.1 ± 7.8 and 95.8 ± 6.7, respectively. In the *pvpA* PCR-HRM, the GCP ± SD value for these strains was 94.9 ± 6.4, 95.0 ± 7.5, 94.2 ± 8.4, 93.3 ± 5.6 and 95.4 ± 5.4, respectively. To calculate the cut off point of GCP for each strain in this experiment, a value of 2 SD was subtracted from the average of respective GCPs. The cut off points in the *vlhA* PCR-HRM when ts-11, 6/85, F, S6 and 86026 used as reference were 79.8, 83.0, 70.8, 78.5 and 82.4, respectively. The cut off points in the *pvpA* PCR-HRM when ts-11, 6/85, F, S6 and 86026 were used as reference were 82.1, 80.0, 77.4, 82.1 and 84.6, respectively. Then calculated cut off points were applied for genotyping mixed-MG strains using PCR amplicons of respective genes. Using cut off value for each MG strain enabled to assess the relationship of other MG strains without visual interpretation by the operator (non-objective). Therefore, all specimens containing mixed MG strains with GCP more than the cut off points of MG strains were genotyped as respective MG strain by the software, while specimens that produced GCPs less than cut off point were automatically genotyped as ‘variation’, indicating mixed strains (not an individual strain).

### Detection of mixed MG specimens using vlhA PCR-HRM

Using each strain as reference genotype, and the cut off point determined for that strain, five DNA mixtures of ts-11 (1 ng) with each of the MG strains F, 6/85, S6 and 86026 at different concentrations (1-10^-4^ ng) were tested in *vlhA* PCR-HRM and GCPs calculated (Table 2). Also mixtures of each contaminant MG strain (1ng) with different concentrations (1-10^-4^ ng) of ts-11 DNA were examined similarly (Table 2).

**Table 2:** GCP values generated using *vlhA* and *pvpA* PCR-HRM from mixed MG strains with different DNA concentration.

Sample	DNA conc (ng)	*vlhA*		*pvpA*	
		Mean GCP	Genotype* (79.8 CP)	Mean GCP	Genotype* (82.1 CP)
ts-11	1	99.9	ts-11	99.1	ts-11
F	1	99.7	F	99.9	F
ts-11 + F	1 + 1	95.2	ts-11	98.5	F
ts-11 + F	1 + 10^–1^	99.6	ts-11	77.2	Variation
ts-11 + F	1 + 10^–2^	99.1	ts-11	90.5	ts-11
ts-11 + F	1 + 10^–3^	99.7	ts-11	98.5	ts-11
ts-11 + F	1 + 10^–4^	99.7	ts-11	98.5	ts-11
ts-11 + F	10^–1^ + 1	91	ts-11	92.9	F
ts-11 + F	10^–2^ + 1	23	Variation	95.0	F
ts-11 + F	10^–3^ + 1	99.1	F	99.0	F
ts-11 + F	10^–4^ + 1	99.7	F	98.9	F
6/85	1	99.4	6/85	99.9	6/85
ts-11 + 6/85	1 + 1	97.8	ts-11	12.1	Variation
ts-11 + 6/85	1 + 10^–1^	97.6	ts-11	34.1	Variation
ts-11 + 6/85	1 + 10^–2^	83.3	ts-11	85.7	ts-11
ts-11 + 6/85	1 + 10^–3^	97.6	ts-11	96.4	ts-11
ts-11 + 6/85	1 + 10^–4^	96.4	ts-11	97.3	ts-11
ts-11 + 6/85	10^–1^ + 1	22.1	Variation	81.3	Variation
ts-11 + 6/85	10^–2^ + 1	32.5	Variation	77.0	Variation
ts-11 + 6/85	10^–3^ + 1	84.3	6/85	97.8	6/85
ts-11 + 6/85	10^–4^ + 1	85.0	6/85	98.0	6/85
S6	1	98.5	S6	97.7	S6
ts-11 + S6	1 + 1	74.5	Variation	81.2	Variation
ts-11 + S6	1 + 10^–1^	71.2	Variation	89.3	ts-11
ts-11 + S6	1 + 10^–2^	46.4	Variation	96.2	ts-11
ts-11 + S6	1 + 10^–3^	99.8	ts-11	99.3	ts-11
ts-11 + S6	1 + 10^–4^	97.4	ts-11	98.8	ts-11
ts-11 + S6	10^–1^ + 1	95.0	ts-11	97.1	S6
ts-11 + S6	10^–2^ + 1	4.6	Variation	95.8	S6
ts-11 + S6	10^–3^ + 1	60.5	Variation	92.8	S6
ts-11 + S6	10^–4^ + 1	99.6	S6	93.5	S6
86026	1	98.5	86026	99.1	86026
ts-11 + 86026	1 + 1	95.1	ts-11	77.3	Variation
ts-11 + 86026	1 + 10^–1^	97.3	ts-11	99.1	ts-11
ts-11 + 86026	1 + 10^–2^	99.5	ts-11	99.9	ts-11
ts-11 + 86026	1 + 10^–3^	98.9	ts-11	99.6	ts-11
ts-11 + 86026	1 + 10^–4^	99.5	ts-11	99.8	ts-11
ts-11 + 86026	10^–1^ + 1	4.4	Variation	26.6	Variation
ts-11 + 86026	10^–2^ + 1	79.4	Variation	16.0	Variation
ts-11 + 86026	10^–3^ + 1	97.3	86026	63.4	Variation
ts-11 + 86026	10^–4^ + 1	99.1	86026	94.3	86026

*variation denotes GCP values under the cut off point when ts-11 and the mixing strain both used as reference. Figures within bracket denote the cut off point applied.

**Fig 1:**
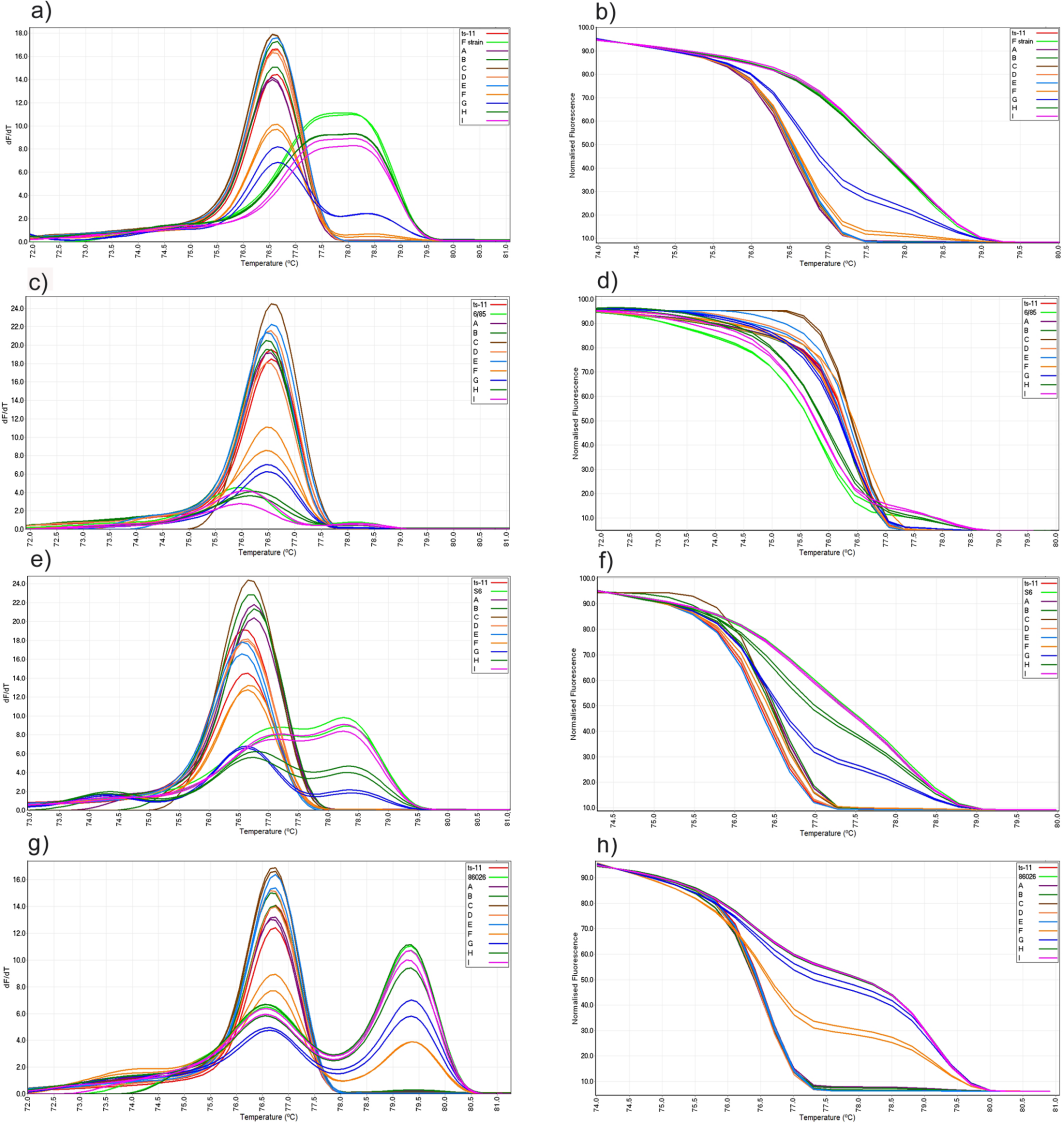
Conventional and normalized melt curve analysis of mixed strains using *vlhA*-PCR. Mixture of ts-11 and F strain (a and b), ts-11 and 6/85 strain (c and d), ts-11 and S6 strain (e and f), ts-11 and 86026 field isolate (g and h). Specimens A, B, C, D and E each contains 1 ng of ts-11 DNA and 1-10^-4^ ng of contaminant MG DNA and specimens F, G, H and I each contains 1-10^-4^ ng of ts-11 DNA and 1 ng of contaminant MG DNA, respectively.

Out of nine specimens containing a mixture of ts-11 at different concentration and 1ng of F strain, only one mixture (10^–2^ ng ts-11) was detected as variation with a GCP of 23 (Table 2). This mixture generated 2 peaks at 76.7 ± 0.01 and 78.4 ± 0.03 in conventional melt curve analysis and a distinct normalized curve compared to ts-11 and F separately (Fig 1A and 1B). The ts-11 or F strain alone were genotyped as ts-11 and F strain respectively.

Mixed specimens of ts-11 and 6/85 strains were genotyped as variation at two out of nine dilutions (10^–1^– 10^–2^ ng of ts-11 and 1 ng of 6/85) with mean GCPs of 22.1 and 32.5, respectively (Table 2). The 2 mixtures each generated 2 peaks in conventional melt curve analysis at 76.2 ± 0.05, 78.1 ± 0.08 and 76.2 ± 0.2 and 78.3 ± 0.3 which were distinct to those of ts-11 and 6/85. These 2 mixtures also produced normalized melt curves which were distinct from those of ts-11, 6/85 and each other (Fig 1C and 1D). All DNA concentrations of 1 ng ts-11 mixed with different DNA concentrations of 6/85 (1-10^-4^) were genotyped as ts-11 with GCPs of ≥ 83.

Five out of nine mixed specimens of ts-11 and S6 strains were detected as “variation” (Table 2). These included three DNA concentrations of 1 ng ts-11 and 1, 10^–1^ and 10^–2^ ng of S6, each of which generated one peak at 76.7 ± 0.4, and two mixtures of 10^–2^ and 10^–3^ ng of ts-11 and 1 ng S6, each of which generated 3 peaks. These five mixtures generated distinct normalized curves to those of ts-11 and S6 (Fig 1E and 1F).

Out of nine mixed specimens of ts-11 and 86026, two mixtures with dilutions of 10^–1^ and 10^–2^ ng of ts-11 and 1 ng of 86026 were genotyped as variation with GCPs of 4.4 and 79.4 (Table 2). These mixtures generated 2 peaks in conventional melt curve analysis at 76.7 ± 0.03 and 79.4 ± 0.01 which were different to those of ts-11 and 86026. The mixtures also produced distinct normalized curves to ts-11 and 86026 (Fig 1G and 1H). All mixed specimens containing 1 ng ts-11 and 1-10^-4^ ng of 86026 were genotyped as ts-11 with GCPs of ≥ 95, while mixtures with lower DNA concentrations of ts-11 (10^–3^ -10^-4^) and 1 ng 86026 were genotyped as 86026 with GCPs of ≥ 97.

### Detection of mixed MG specimens using pvpA PCR-HRM

**Fig 2:**
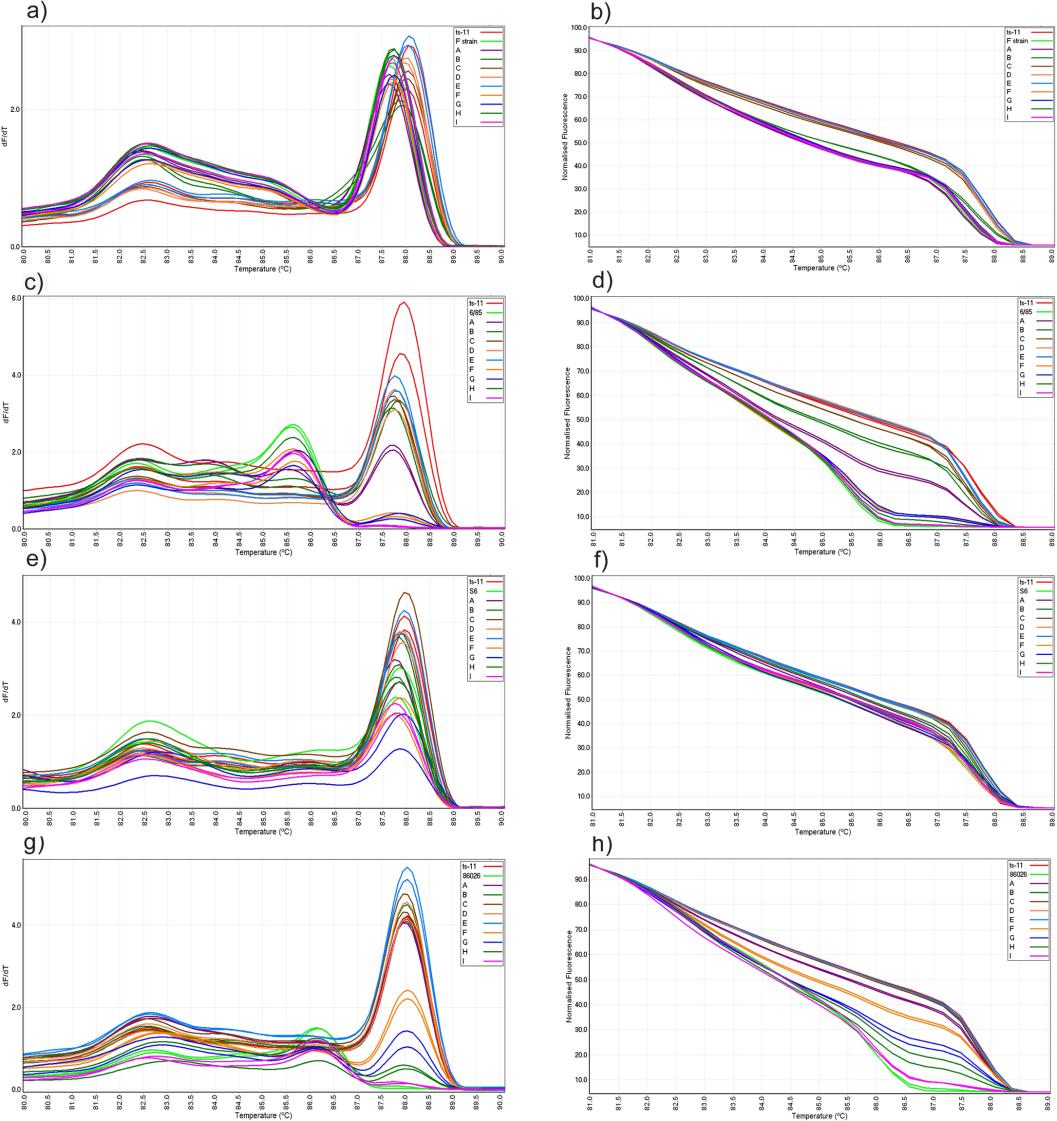
Conventional and normalized melt curve analysis of mixed strains using *pvpA*-PCR. Mixture of ts-11 and F strain (a and b), ts-11 and 6/85 strain (c and d), ts-11 and S6 strain (e and f), ts-11 and 86026 field isolate (g and h). Specimens A, B, C, D and E each contains 1 ng of ts-11 DNA and 1-10^-4^ ng of contaminant MG DNA and specimens F, G, H and I each contains 1-10^-4^ ng of ts-11 DNA and 1 ng of contaminant MG DNA, respectively.

Nine mixed dilutions of ts-11 and each of the MG strains F, 6/85, S6 and 86026, were examined in *pvpA* PCR-HRM using a cut off point determined for that strain.Only one specimen out of nine mixtures containing ts-11 and F strain was detected as variation. This specimen was a mixture of 1 ng ts-11 and 10^–1^ ng of F strain with a mean GCP of 77.2, and produced two peaks at 82.5 ± 0.1 and 87.9 ± 0.1 in conventional melt curve analysis and distinct normalized curve to those of ts-11 and F strains. Three DNA mixtures containing 1 ng of ts-11 and 10^-2^-10^-4^ ng of F strain were genotyped as ts-11 and generated 2 peaks similar to ts-11 with GCPs of ≥ 90 (Table 2). One DNA mixture containing 1 ng of ts-11 and 1 ng of F strain and four mixtures of 10^-1^-10^-4^ ng of ts-11 and 1 ng of F strain were all genotyped as F strain and generated 2 peaks similar to F strain with GCPs of ≥ 92 (Fig 2A and 2B).

Out of nine mixed specimens of ts-11 and 6/85, four mixtures were genotyped as variation. These included two mixtures of 1 ng of ts-11 with 1 and 10^–1^ ng of 6/85 which generated mean GCP of 12.1 and 34.1 respectively, and two dilutions of 10^–1^ and 10^–2^ ng ts-11 with 1 ng of 6/85 which generated mean GCPs of 81.3 and 77.0 respectively (Table 2). These mixtures each generated 4 peaks at 82.8 ± 0.4, 83.8, 85.5 ± 0.2 and 87.7 ± 0.1 in conventional melt curve analysis and distinct normalized curves to those of ts-11 and 6/85 (Fig 2C and 2D). Three mixtures of 1 ng ts-11 and 10^-2^-10^-4^ ng 6/85 were genotyped as ts-11 while two mixtures of 10^-3^-10^-4^ ng of ts-11 and 1 ng of 6/85 were genotyped as 6/85.

Only one mixture of ts-11 (1 ng) and S6 (1 ng) was detected as variation (Table 2). This mixture produced one peak at 87.8 ± 0.1 in conventional melt curve analysis and distinct normalized curve to those of ts-11 and S6. Four mixtures containing 1 ng of ts-11 and 1^-1^-10^-4^ ng of S6 were genotyped as ts-11 with GCPs of ≥ 89 and four mixtures of 10^-1^-10^-4^ ng of ts-11 and 1 ng of S6 were genotyped as S6 with GCPs of ≥ 92.

Mixed specimens of ts-11 and 86026 were genotyped as variation at four out of nine dilutions including one mixture containing equal DNA concentrations of 1 ng ts-11 and 1 ng 86026, and three mixtures containing 1 ng of 86026 and 10^–1^, 10^–2^ and 10^–3^ ng of ts-11 (Table 2). These mixtures each generated 3 peaks at 82.9, 86.0 and 88.1 in conventional melt curve analysis and distinct normalized curves to those of ts-11 and 86026 (Fig 2G and 2H). All DNA mixtures containing 1 ng of ts-11 and 10^–1^ -10^-4^ ng of 86026 were genotyped as ts-11 and the mixture of 10^–4^ ng ts-11 and 1 ng 86026 was genotyped as 86026.

### Comparison of vlhA PCR-HRM and pvpA PCR-HRM curve analysis in their potential to detect mixed MG specimens

The performance of the *vlhA* and *pvpA* PCR-HRM curve analysis is summarized in Table 3. In brief, *vlhA* PCR-HRM genotyped 19 mixed specimens as ts-11, 7 as the mixing (contaminant) strain and 10 as variation. In contrast, *pvpA* PCR-HRM genotyped 14 mixed specimens as ts-11, 12 as the mixing (contaminant) strain and 10 as variation. Although the number of mixed specimens detected as variation was similar in both PCR-HRMs, *vlhA* PCR-HRM showed more predilection for ts-11 genotype while *pvpA* PCR-HRM showed more predilection for the mixing strain genotype.

**Table 3:** Number of specimens detected as ts-11, mixing strain (F, 6/85, S6 or 86026), or “variation” in *vlhA* and *pvpA* PCR-HRM.

Genotype detected	PCR-HRM used	
	*vlhA*	*pvpA*
ts-11	19	14
mixing strain	7	12
variation	10	10
total	36	36

The percentage of sequence identity in PCR amplicons of MG strains was also compared (Table 4). The highest and lowest sequence identity in *vlhA* gene was observed between F and S6 and 6/85 and 86026 MG strains, respectively. The highest and lowest sequence identity in *pvpA* gene was observed between 6/85 and S6 and 6/85 and 86026 MG strains, respectively.

**Table 4:** Percentage sequence identity of MG strains in *vlhA* and *pvpA* genes.

	Percent identity in *vlhA* gene				
MG strain	ts-11	6/85	F	86026	S6
ts-11	-	56.4	79.2	71.2	81.0
6/85	94.4	-	55.7	37.6	56.4
F	95.7	59.8	-	92.2	98.7
86026	96.5	49.2	93.6	-	92.2
S6	95.4	99.2	90.6	92.0	-
	Percent identity in *pvpA* gene				

### Further evaluation of PCR-HRM curve analysis using *vlhA* and *pvpA* genes for identification of mixed MG strains in clinical swabs

**Fig 3:**
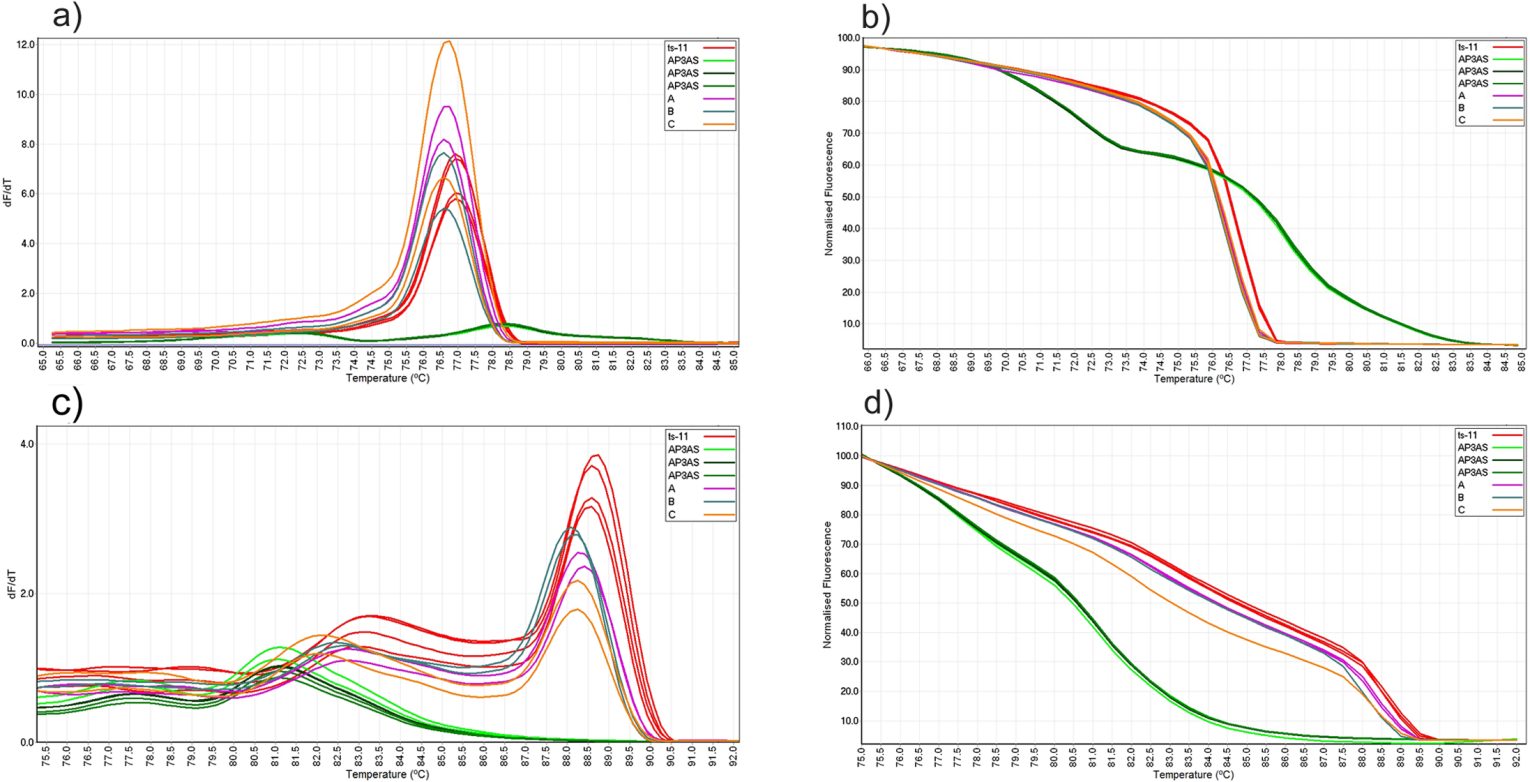
Conventional and normalized melt curves analysis of PCR products of *vlhA* (a and b) and *pvpA* (c and d) genes from choanal swab samples. Mixed specimens A, B and C contain equal DNA concentrations (1 ng) of ts-11 and AP3AS strains.

Mixed DNA samples of ts-11 and AP3AS DNA from choanal swabs were tested using *vlhA* and *pvpA* PCR-HRM and the cut off values established for each PCR applied. In both *vlhA* and *pvpA* PCR-HRMs, all mixed specimens produced conventional and normalized curves which were distinct from those of ts-11 and AP3AS (Fig 3). The GCPs produced by mixed specimens were <59 and <73 in *vlhA* and *pvpA* PCR-HRMs respectively which were less than the cut off points established for these PCRS, and therefore all mixed specimens were automatically genotyped as “variation”.

## Discussion

The *vlhA* and *pvpA* PCR-HRM tests described in this study were developed to evaluate and compare the potential of HRM curve analysis to detect mixed MG strains in a specimen. Since the ts-11 vaccine strain is widely used in poultry flocks around the world, we used a combination of ts-11 and other MG strains as a model to evaluate this potential.

Different tests for differentiation of MG strains/isolates have been developed [11–13, 16]. The increasing uses of the live MG vaccines such as ts-11, F or 6/85 in poultry farms has necessitated development of laboratory tests that can differentiate these vaccine strains from field isolates. A number of molecular methods have been developed and reported to differentiate MG strains in a mixed population [17–20]. The PCR-HRM has been used to differentiate individual avian pathogens including MG strains [13, 21]. The capacity of HRM curve analysis for differential detection of pathogenic *Eimeria* species of chickens has been evaluated [25]. However, the capability of PCR-HRM to detect mixed strains of a particular organism in a single specimen has never been examined.

Both PCR-HRM curve analysis were capable of detecting (as “variation”) ten of the mixed MG specimens (ts-11 and F, 6/85, S or 86026). Comparing the nucleotide variability of tested MG strains for *vlhA* and *pvpA* genes, slightly higher nucleotide sequence variability was observed between ts-11 vaccine strain and MG strains in *vlhA* gene nucleotide sequences [21]. However, this was not reflected in higher capacity of *vlhA*-HRM for detection of mixed specimens compared to *pvpA*-HRM. High GC content (>60%) can also be a critical factor in differentiation power of a PCR-HRM system [26] but the *vlhA* and *pvpA* amplicons all had a GC content of less than 50%. The amplicon size also plays a crucial role in HRM curve analysis [27]. Amplicon sizes of 200–400 bp (or less) can increase the detection sensitivity of sequence diversity in tested specimens [28, 29]. The amplicons for the *vlhA* gene from tested MG strains were smaller than those of *pvpA*. It has been reported that higher nucleotide sequence diversity in *vlhA* gene along with smaller PCR amplicon sizes compared to *pvpA* gene PCR amplicons were responsible for better performance of *vlhA* PCR-HRM in identifying individual MG isolates [21]. However, this was not observed when mixed MG specimens were tested. This could be due to co-amplification of the mixing MG strain and its contribution to the shape of HRM curve generated. It is believed that the quality of DNA may potentially affect HRM curves but extraction of DNA was consistent across all MG strains in this study and therefore quality of DNA is less likely to contribute to variations in melt curves seen here.

Irrespective of the number of “variations” detected, the *vlhA* PCR-HRM appeared to show a higher tendency to genotype mixed specimens as ts-11 while *pvpA* PCR-HRM appeared to show a higher tendency to genotype mixed specimens as the strain mixed with ts-11. This is perhaps expected since primers used in *vlhA* PCR-HRM were originally designed based on sequences available for ts-11 [13]. If an alternative strain (other than ts-11) had been used as the base strain, it is possible that the *pvpA* PCR-HRM could have performed more satisfactorily.

The PCR-HRM results in this study are entirely based on using extracted DNA from pure MG cultures. Limited experience in our laboratory suggest that infection of ts-11 vaccinated chicken flocks with a field strain could be detected using HRM curve analysis if the second strain was present at high levels and a large number of birds examined (results not shown). Therefore the success of the HRM test is in accordance with the quantity and proportion of DNA of the infecting field strain in the specimen.

Results from this study also suggested that a higher number of variations detected in the *vlhA* PCR-HRM when ts-11 and S6 (as opposed to ts-11 and F strain) were present in a mixture. For the *pvpA* PCR-HRM, the highest number of variations were detected when ts-11 and 6/85 or 86026 were present in the specimen. This is expected as differentiation power of PCR-HRM analysis relies heavily on sequence diversity of tested genes in the specimens. Thus, it is stipulated that PCR-HRM technique for detection of mixed MG vaccine strain and field isolates might be more applicable in countries where the variability of *vlhA* and *pvpA* genes of the field MG isolates is high. This can be readily predicted by analysing *vlhA* and *pvpA* gene sequences of a range of field isolates from that region.

The *vlhA* and *pvpA* PCR-HRM technique in this study each could detect some specimens containing mixed MG strains, however a combination of *vlhA* and *pvpA* PCR-HRM could potentially increase the capacity for detection of mixed specimens. Alternatively, detection of two or more MG strains in a single specimen could be improved perhaps if HRM curve analysis is combined with strain specific probes.

The number and the melting point of the peaks, and the shape and height of HRM curves are as a result of nucleotide variations in DNA sequences of MG strains and therefore could be unique for each strain/isolate [13]. This test is most useful for screening of clinical specimens collected from flocks vaccinated with a live *M*. *gallisepticum* vaccine, as these flocks may hypothetically contain a field strain in addition to the vaccine strain used. Both strains can contribute to the shape of conventional and normalized curves. This however should be readily detectable given that the melting curve profile of all the existing *M*. *gallisepticum* vaccine strains are already characterized. Where “variation” is detected in clinical specimens from such flocks, the presence of the second strain is best to be confirmed using additional tests such as PCR followed by nucleotide sequencing of the amplicons [30].

The potential of PCR-HRM using *vlhA* and *pvpA* genes was further evaluated by testing mixed DNA samples from clinical swabs obtained from experimentally inoculated turkeys and chickens. All mixed samples were differentiable from ts-11 and AP3AS MG strains in both PCR-HRM techniques.

The results presented here highlights that PCR-HRM curve analysis can be used as a screening tool for monitoring of suspected mixed MG infections particularly in samples submitted from vaccinated flocks. The test results could be revealed in less than eight hours which can contribute to more timely and efficient MG control measures.
